# Integrated hypothesis of dental caries and periodontal diseases

**DOI:** 10.1080/20002297.2019.1710953

**Published:** 2020-01-07

**Authors:** Bente Nyvad, Nobuhiro Takahashi

**Affiliations:** a Section of Dental Pathology, Operative Dentistry and Endodontics, Department of Dentistry and Oral Health, Aarhus University, Aarhus, Denmark; b Division of Oral Ecology and Biochemistry, Department of Oral Biology, Tohoku University Graduate School of Dentistry, Sendai, Japan

**Keywords:** Dental caries, periodontal diseases, ecosystem, microbiota, carbohydrates, gingival crevicular fluid, resilience factor, dynamic stability stage, ecological hypothesis

## Abstract

This review considers an integrated hypothesis of dental caries and periodontal diseases that builds on theoretical ecological principles. The backbone of the hypothesis is based on the dynamic stability stage of the oral microbiota, at which intrinsic (mainly saliva and gingival crevicular fluid) and bacterial (mainly metabolic) resilience factors maintain ecological dynamic stability, compatible with clinical health. However, loss of intrinsic resilience factors and/or prolonged changes in the availability of microbial metabolic substrates may shift the ecological balance of the microbiota into either saccharolytic (acidogenic) or amino acid-degrading/proteolytic (alkalinogenic) stages, depending on the nature of the predominant substrates, leading to clinical diseases. Therefore, to maintain and restore the dynamic stability of the oral microbiota, it is necessary to control the drivers of disease, such as salivary flow and influx of bacterial nutrients into the oral cavity. Contrary to conventional wisdom, excessive intake of fermentable carbohydrates may contribute to inflammation in periodontal tissues resulting from hyperglycaemia. An integrated hypothesis emphasizes that both dental caries and periodontal diseases originate in the dynamic stability stage and emerge in response to nutritional imbalances in the microbiota. Periodontal diseases may belong to the sugar driven inflammatory diseases, similar to diabetes, obesity, and cardiovascular diseases.

## Introduction

Over the years, considerable effort has been invested in explaining and understanding the etiology of the major dental diseases, caries and periodontal diseases. Early experimental animal studies promoted specific bacteria as the cause of dental diseases [1], but this hypothesis was later rejected because no single bacterium among the oral microbiota could be held responsible for disease development [2]. Meanwhile, Koch´s classical definition of specificity was amended to allow groups of bacteria to satisfy the definition of microbial specificity [3,4]. Rather than being facilitated by single ‘pathogenic’ species, dental diseases were recognized as an overgrowth of particular members of the indigenous microflora [5,6]. It was only in 1994 that the ecological plaque hypothesis was formally formulated [7]. According to the ecological hypothesis, changes in the environment can predispose a site to disease because of modifications in the composition of the resident microflora. This ecological concept is now the prevailing hypothesis to understanding the circumstances associated with the development of caries and periodontal diseases [8–15].

More recently, the ecological hypothesis was extended and refined to highlight the overriding importance of the metabolic activity of the oral microbiota – rather than its composition – as the principal modulator of the environment [13–17]. Microbial changes and the subsequent modification of the pathogenic properties of the microbiota were explained as responses to ‘regime shifts’ in which one metabolic stage of the microbiota leads to new stages due to changes in local nutritional and immunological environments. Regime shifts are well known in a range of biological ecosystems [18]. In this philosophy, the oral bacteria do not select their own habitat because of particular pathogenic traits – they are selected by an environment they partly create themselves.

An important tenet of the extended ecological hypothesis rests on the dynamic metabolic activities occurring in the microbiota on any tooth surface at any time in the oral cavity. These processes are reflected by constant fluctuations of pH in the dental biofilm, both when the microbiota is starved and when fed, and are considered to be natural physiological phenomena [19]. Most of the time, the continuous pH-fluctuations are balanced over time and do not give rise to clinical disease because of compensatory reactions in the microbial community. We have named this particular stage of equilibrium the ‘dynamic stability stage’ [14,15]. At this stage pH of the microbiota fluctuates between pH 5.5–7. However, from time to time more pronounced ecological pressures of dietary, salivary and/or inflammatory nature might cause a drift of the pH-balance. Such drifts in environmental pH could predispose a site to dental caries and/or gingivitis, respectively, depending on the direction of the pH-shift.

Previous authors have reviewed the various ecological interactions in oral health and disease [20, 21, 22, 23], while Mira et al. [24], and Manji et al. [19], speculated about a common mechanism for the development of caries and periodontal diseases. However, so far no authors have been able to convey an all-encompassing theory to substantiate or explain systematically such speculations [25]. The aim of this review is to consider an integrated hypothesis for caries and periodontal disease and to discuss the physiological and ecological principles behind this hypothesis. In particular, we wish to highlight the functional mechanisms that confer stability or resilience [23] to the dynamic stability stage of the microbiota on teeth. If we can define and explain the functions that stabilize, disrupt and restore the oral microbial ecosystem, this might lead to improved ecological approaches on how to control or modulate disease development in the future. Finally, we argue that an integrated hypothesis is compatible with a hypothesis suggesting that excess intake of fermentable carbohydrate is a shared risk factor of dental caries and periodontal diseases.

## Physiological dynamics of the oral microbiota and its modulation in the oral environment

**Figure 1.**
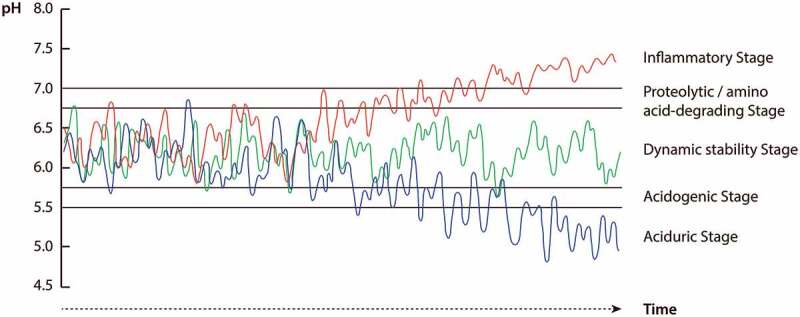
Illustration of three theoretical pH response patterns in the integrated hypothesis of dental caries and periodontal diseases. The green curve illustrates pH fluctuations of the microbiota at the dynamic stability stage associated with clinical health, at which pH fluctuations are balanced over time due to infrequent nutrient exposures. The red and blue curves show pH fluctuations of the microbiota at subgingival and supragingival sites in response to increasing exposure microbial nutrients, respectively. When GCF/inflammatory nutrients are intense and prolonged, pH of the microbiota may drift from the dynamic stability stage into proteolytic/amino-acid degrading and inflammatory stages associated with gingivitis and periodontitis (red curve), whereas intense and prolonged increases in exposure to dietary carbohydrates promotes a drift of pH into acidogenic and aciduric stages associated with caries (blue curve). For further details, see text

The functional core of the oral microbial ecosystem is the dynamic stability stage at which teeth, gingivae and oral mucosal surfaces remain clinically sound, in spite of the presence of dental plaque. Figure 1 represents a graphic illustration of different scenarios associated with health and disease in the oral ecosystem. The figure integrates theoretical profiles of pH in the supra- and subgingival microbiota according to various stages of the extended ecological hypotheses [14–16]. At the dynamic stability stage, the microbiota comprises a diverse microbial community dominated by non-mutans streptococci and *Actinomyces*, the resting pH of which fluctuates between pH 5.5–7.0, with a mean pH of 6.25 in healthy individuals [26]. These pH-fluctuations are partly caused by continuous microbial metabolism of host-derived nutrients from saliva and gingival crevicular fluid (GCF), and partly by metabolism following intermittent intake of foods. At this stage, it cannot be avoided that plaque-pH occasionally changes temporarily to lower or higher values, but in the healthy individual salivary and GCF clearance mechanisms together with compensatory bacterial metabolic activity of the microbiota itself, may easily restore the pH-balance. Under these conditions, it is unlikely that the dental hard tissues may suffer clinically relevant mineral losses, as environmental pH seldom surpasses the ‘critical’ value for dissolution of hydroxyapatite for extended periods of time.

However, if the pH-balance of the microbial community is disrupted by severe environmental pressures, such as increases in the frequency and concentration of carbohydrate consumption, the normal homeostatic reactions conferred by saliva and the commensal microflora may collapse, and the pH-balance drifts into the ‘acidogenic stage’ (Figure 1). This environmental shift is caused by acid adaptation and acid selection of bacteria, such as low-pH non-mutans streptococci that are more suited to live an acidic habitat fluctuating around pH 5.5–6 [27–29]. At this stage, an increase in the frequency and duration of average pH around pH 5.5 might, other things being equal, initiate demineralization processes of the dental hard tissues.

Eventually, in the case of a prolonged and intense carbohydrate supply, the microbial community pH might shift to average values fluctuating between pH 4.5–5.5 [26] and enter the ‘aciduric stage’ (Figure 1). At this stage, aciduric specialists capable of metabolizing and surviving at particularly low pH environments [fx mutans streptococci and non-mutans aciduric bacteria including strains of *Actinomyces* and bifidobacteria) are selected among the resident community members to partly replace the less aciduric species. The major principles of these acid adaptation and acid selection processes in response to increased carbohydrate exposure have been described in detail by Takahashi [13], and Takahashi and Nyvad [14,15]. It should be appreciated that similar and more abrupt changes in the ecology of the microflora could occur in response to reduction in the rate of salivary secretion. In the latter case, a relative increase of the nutrient supply stems from reduced oral clearance.

The pH-balance at the dynamic stability stage could also be driven towards higher pH-values. This is likely to happen in response to gingivitis. Gingivitis is the host inflammatory response to microbial challenge, which if left unattended, may lead to breakdown of host-microbe homeostasis and degradation of the periodontium [for review, 11, 30]. Gingivitis increases the secretion of a serum-like exudate (GCF] in the gingival crevice [31]. This protein-rich environment enhances the growth of indigenous proteolytic and amino acid-degrading bacteria, such as *Fusobacterium* and *Prevotella*, the alkaline cytotoxic products of which raise the average pH to around neutral values. At this stage the microbial community enters the ‘proteolytic and amino-acid degrading stage’ (Figure 1). The major principles of this vicious cycle have been described in detail by Takahashi [13,16]. Continued proteolysis along with acid neutralization by *Fusobacterium* and *Prevotella* [32] may help to generate an alkaline environment for the succession of more acid sensitive ‘inflammophilic’ species, such as *Porphyromonas gingivalis* [33]. In this scenario, *Prevotella intermedia* plays an important modulatory role because it can live in both a sugar-rich supragingival environment and in a protein-rich, neutral to weakly alkaline subgingival environment; yet its cytotoxic and proteolytic virulence is increased only when sugar is absent, such as in subgingival sites with restricted access to dietary sugars [34].

Gingival inflammation is supposed to be the driver for the conversion of gingivitis to periodontitis although the host-immune response is likely to play a significant role in regulating the outcome of the pathological processes [for review, 35,10]. In the subgingival pocket, enhanced inflammatory exudates and bleeding offer a nutritious, weakly alkaline environment – ‘the inflammatory stage’ – suited for the emergence of ”inflammophilic” anaerobic microorganisms, such as *P. gingivalis, Tannerella* and *Treponema* (Figure 1). *P. gingivalis* is attracted to this niche because it exploits the hemin contained in blood hemogloblin for growth, while the growth of some oral *Treponema* species depends on short-chain fatty acids excreted from other bacteria [13]. Moreover, *P. gingivalis* exploits the slightly alkaline environment in the subgingival pocket (pH 7–7.5) [36] to become dominant [32]. Importantly, acidogenic taxa, such as *Streptococcus* and *Actinobacteria*, are also common members of the microbiota at sites with periodontal disease [8,37]. Co-colonization of these species with acid neutralizing species, such as *Fusobacterium* and *Prevotella* [16], may therefore confer an important ecological advantage to *P. gingivalis* by sustaining an alkaline environment in the periodontal pocket. It follows from this that shifts in the composition of the microflora associated with the development of gingivitis and periodontitis are the result of bacterial degradation of host proteins from gingival inflammatory exudates followed by microbial selection induced by the metabolically modified environment. Hence, inflammation is the primary driver of the development of periodontal disease, and not the presence of specific microbial species, although periodontitis-associated bacteria such as *P. gingivalis* and oral treponema can aggravate disease severity by enhancing protein/amino acid metabolism and host inflammation.

The above description (Figure 1) lends support to a theoretical framework for an integrated hypothesis of microbial dental diseases based on a continuous scale of dynamic stages. The major determinants in this microbial ecosystem are dietary and/or host-derived nutritional factors, which have a constant modulatory effect on pH and microbial ecology of the community. The core of the ecosystem is the dynamic stability stage at which the dental microbial community is stable and balanced over time due to its ability to control disturbances by external factors [38]. This ecological stage is compatible with clinical health. Temporary perturbation of the community balance can be restored and eventual consequences restricted to subclinical signs of disease. Therefore, from a theoretical perspective, there is no such condition as pristine dental health [39,19]. As metabolic microbial processes occur incessantly in the dental plaque, subclinical signs of dental diseases cannot be prevented, but their clinical manifestations can be controlled.

To fully appreciate the concept of dynamically stable oral environments it is helpful to review some basic ecological phenomena from environmental biology, such as resilience, biodiversity and regime shifts [18,40].

## The concept of resilience

A cornerstone of steady states in biological ecology is resilience. The current best definition of resilience is the ‘capacity of an ecosystem to absorb disturbance and reorganize while undergoing change so as to still retain essentially the same function, structure, identity and feed backs’ [41]. A crucial aspect of the definition is the degree to which a given ecosystem can reorganize and renew itself after disturbance [42,43]. Inability to cope with environmental change – i.e. loss of resilience – can destabilize previously stable communities and turn them into new stability domains, referred to as regime shifts [18,44]. The integrated hypothesis for caries and periodontal disease proposed in this paper is compatible with the ecologic thinking of a continuum of dynamic states [18]. In this concept, the ‘dynamic stability stage’ [14,15], holds the characteristics of a dynamic steady state controlled by resilience.

Biological diversity plays a significant role in ecosystem resilience and in sustaining desirable ecosystem states in the face of change [45]. The ability of an ecosystem to perform compensatory community benefits has been referred to as the ‘insurance hypothesis’ because of the idea that greater species richness insures the ecosystem against declines in functioning if some species fail [46]. However, taxonomic diversity *per se* does not guarantee stability. Diversity of functional groups and the functional diversity among species within these groups could have a stronger impact on resilience [47,48]. Elmqvist and co-workers [49] referred to the latter property as ‘response diversity’. Ecosystems, irrespective of their composition, that lack adequate adaptive functional responses to environmental perturbation – i.e. resilience – are functionally debilitated and may undergo regime shift to a different stability domain [43].

## Resilience factors at the dynamic stability stage

Resilience of the oral microbiome has previously been discussed by [23]. Rosier and co-workers described comprehensive synergistic and antagonistic reactions including bacterial metabolism and host immune reactions that may lead to symbiosis and dysbiosis in the microbiota. Symbiosis is a descriptive term meaning the status of ‘living together in harmony with the host’. However, the term symbiosis does not explain the functions that generate and/or maintain this status. Symbiosis can be achieved by dynamic stability provided by the functions of resilience factors. Hence, the dynamic stability stage is a function-focused term of symbiosis; accordingly, symbiosis is a status provided by dynamic stability.

**Figure 2.**
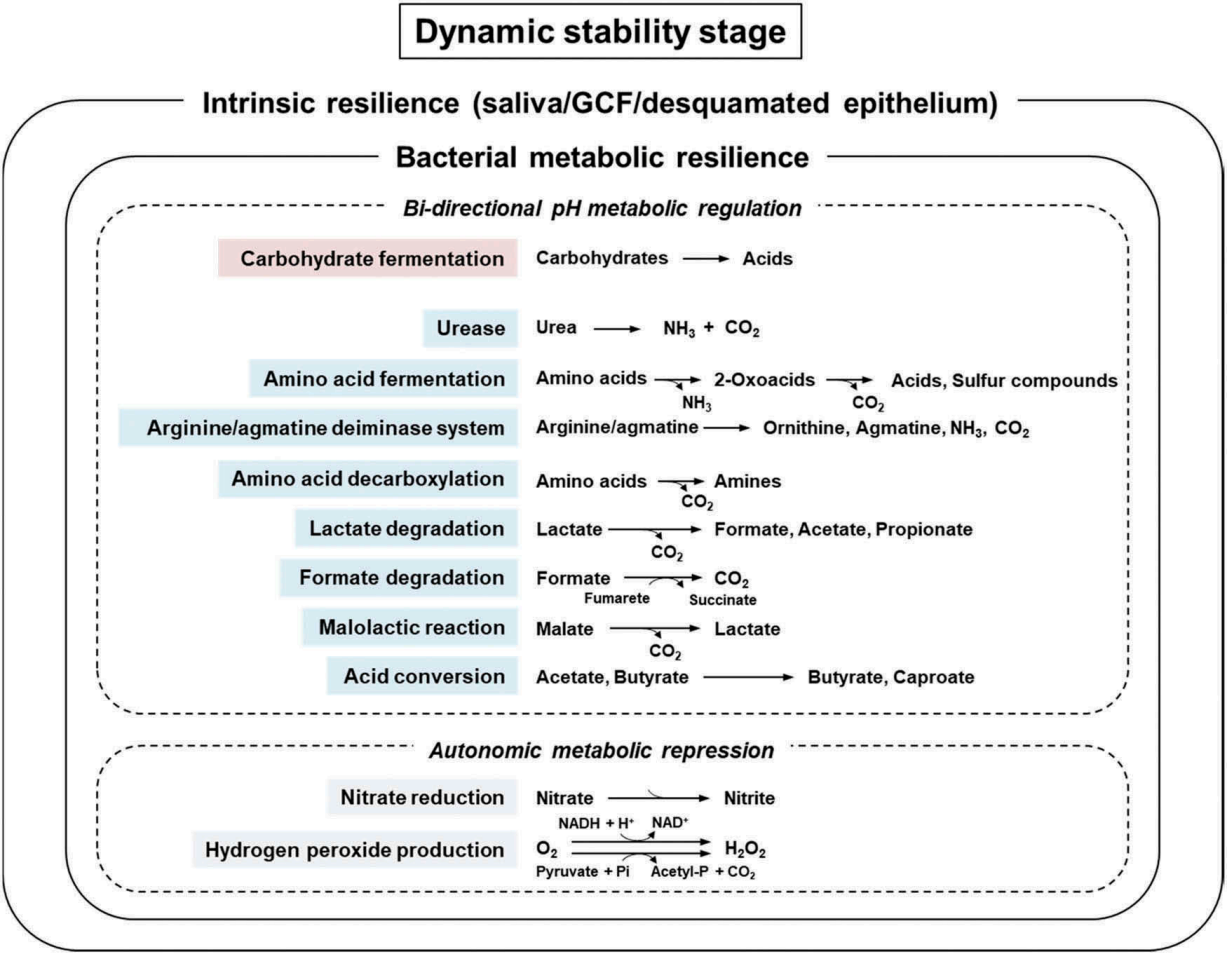
Overview of intrinsic and bacterial metabolic resilience factors operating at the dynamic stability stage. Bacterial metabolic resilience comprises two categories: bi-directional pH metabolic regulation and autonomic metabolic repression. Resilience factor marked in red = acidification factor, which is more active under alkaline than under acidic conditions. Resilience factors marked in blue = acid-neutralization/alkalization factors, some of which are more active under acidic than under alkaline conditions or active at a wide range of pH. Some bacterial metabolites, such as NH_3_ and sulfur compounds are inflammatory factors. For detailed description, see text

In view of the important role of bacterial metabolic resilience in maintaining dynamically stable microbial communities, we have identified the resilience factors that come into play at the dynamic stability stage [16]. According to our understanding, intra-oral resilience at the dynamic stability stage can be divided into two categories; those conferring *intrinsic resilience*, such as saliva, GCF, and desquamated epithelium, and those conferring *bacterial metabolic resilience* (Figure 2). Bacterial metabolic resilience further comprises two types; bi-directional pH metabolic regulation and autonomic metabolic repression.

### Intrinsic resilience

Saliva plays a key role in maintaining oral health because of its clearance mechanisms of food and acids from the mouth [50, 51]. This is why saliva is regarded as the most important resilience factor in the oral cavity. It is well known that patients with decreased salivary secretion develop an aciduric microbiota [52] and suffer more caries [53] than patients with normal salivary flow because of poor bicarbonate buffering of pH and reduced oral clearance. At the same time, saliva safeguards the survival of the oral microorganisms between meals by providing nutrients such as glycoproteins, proteins, urea and minerals for bacterial growth. Subgingivally, GCF containing serum-like constituents (proteins, amino acids) deliver the basic nutrients for bacterial growth, together with urea [54] and keratin-like proteins derived from desquamated gingival epithelia. Collectively, these host-derived factors, including nutrients and the buffered neutral environmental pH offered by saliva and GCF, create a relatively stable environment supra- and subgingivally that is able to counteract minor environmental perturbations induced by the host and the microbiota. Moreover, efflux of small amounts of GCF from the healthy gingival crevice [31] partially inhibits the influx of saliva, food and drink.

Antimicrobial factors, such as immunoglobulins, antimicrobial peptides and enzymes in saliva and GCF, and innate immune systems such as Toll-like receptors in oral mucosa, are also included as host resilience factors. Finally, the host’s response to inflammation plays a pivotal role for the outcome of the host-parasite interactions in periodontal tissues [10].

### Bacterial metabolic resilience

Bacterial metabolic resilience is the pH-modulatory functions that are generated by bacterial metabolic activities.

#### Bi-directional ph metabolic regulation

The pH-modulatory functions are acidification, alkalization or neutralization of pH. The typical modulatory biochemical pathways are listed in Figure 2 [adapted from 16], in which acidification factors are marked in red and acid-neutralization/alkalization factors are marked in blue. In general, the bacterial acid producing activity is relatively high under neutral pH to weakly alkaline conditions, while it decreases as the pH becomes more acidic. Conversely, the acid-neutralizing and alkalizing metabolic activities of amino acid fermentation [33], the arginine deiminase system [55], and amino acid decarboxylation [56] are known to be high at acidic pH. This bi-directional pH metabolic regulation is probably a major bacterial metabolic resilience factor and seems to function complimentarily and automatically to maintain the environmental pH within the 5.5–7 range in the supragingival plaque at the dynamic stability stage.

During periods between meals saliva acts as the main nutritional source for microorganisms in the supragingival plaque. Salivary glycoproteins can be deglycosylated by several glycosidyses from saliva and oral bacteria. Saccharolytic bacteria can subsequently utilize deglycosylation-derived sugars and produce acids, while proteolytic bacteria and salivary proteases [17] can break down deglycosylated glycoproteins into peptides and amino acids. These amino acids as well as amino acids that are normally present in saliva can be further metabolized via various pathways, and help to counteract bacterial acidification (see pathways marked in blue in Figure 2).

Some common bacteria in the supragingival plaque, such as *Actinomyces* and *Streptococcus*, possess urease [16], which quickly breaks down urea into ammonia and carbon dioxide, and neutralizes bacteria-derived acids. Bacterial urease can function at a wide range of pH while keeping high activity at acid pH [57]. Other metabolic pathways, such as bacterial amino acid fermentation, the arginine/agmatine deiminase system and amino acid decarboxylation are also known to neutralize environmental pH (Figure 2) [16]. In addition, bacteria-derived acids can be degraded or converted into weaker acids via lactate degradation, formate degradation, malolactic reaction, and acid conversion (Figure 2) [16]. These acidification and acid-neutralization/alkalization processes that occur repeatedly in the supragingival plaque counteract each other and limit major pH-fluctuations exceeding the 5.5–7 range between meals.

In the subgingival pocket the bacteria gain their primary nutrients from GCF, including immune-related proteins, such as complement proteins and immunoglobulins, and desquamated epithelia. The supply of exogenous carbohydrates is limited due to continuous efflux of GCF. In this anaerobic ecological niche, bacterial amino acid fermentation is the main metabolic bacterial resilience factor that aids in maintaining a neutral environmental pH because of simultaneous production of organic acids and ammonia. Proteolytic bacteria, such as *Prevotella* and *Porphyromonas*, break down and utilize host-derived proteins as nitrogenous substrates for growth. This stage of dynamic stability can be maintained as long as the relationship between the host and the microbiota is balanced. However, excess bacterial proteolytic activity, e.g. due to bacterial accumulation and/or bacterial adaptation to a GCF-rich environment [34] can be cytotoxic and aggravate tissue inflammation by modulating host-immune responses [58] and promoting apoptosis [59].

It should not be ignored that the microbiota on oral mucosal surfaces can also show bacterial metabolic resilience. Especially the tongue surface can be important as a refuge for microorganisms before tooth eruption and in the edentulous mouth. On the surfaces of the oral mucosa and tongue, saliva and desquamated epithelia are the main metabolic substrates, and therefore it is likely that the environment surrounding these soft tissues is somewhat similar to that of the supragingival environment. However, the complex papillary anatomy of the tongue surface, especially at the back of the tongue, encourages accumulation of relatively thick bacterial deposits and desquamated epithelia [60]. This anaerobic protein-rich ecological niche favors the colonization of *Porphyromonas* and *Prevotella* [32] and oral malodor-associated bacteria such as *Fusobacterium nucleatum* [61]. There is still limited information about microbial community shifts in tongue coatings and potential driving forces. Both dietary carbohydrates and amino acid/peptide/protein are regularly supplied as microbial nutrients, while saliva washes the surface of the tongue as an intrinsic resilience factor. Together with continuous shedding of bacteria-coated epithelial cells, this might explain the neutral resting pH values (pH 7) recorded on the tongue surface [26]. Moreover, an extended pH recovery phase following sucrose rinse on the tongue has been ascribed to the lack of an underlying mineralized tissue [26]. In this environment, *Veillonella* species, which are lactate-utilizing and acid-tolerant anaerobes, can become dominant. *Veillonella* species are also eminent producers of hydrogen sulfide in the presence of lactate at acidic pH [62].

#### Autonomic metabolic repression

Bacterial metabolism can also convert host-derived components into active reagents, some of which might regulate bacterial activity. Oral indigenous bacteria, such as *Veillonella* and *Actimomyces*, are known to reduce nitrate (NO_3_^−^), a component of green vegetables, into nitrite (NO_2_^−^) [63]. While in the oral cavity, nitrite inhibits bacterial acid production from carbohydrates [64,65]. Nitrite is subsequently absorbed into the host´s blood circulation (where it participates in blood pressure control) before it is gradually oxidized and converted back to nitrate and expelled in urine; however, about 10% of the nitrate is recirculated and secreted into the oral cavity via saliva. This is called the ‘enterosalivary circulation’ [66].

A classic, but complicated example of a host-derived metabolic pathway is the salivary peroxidase-hypothiocyanate system. Hypothiocyanate is secreted from saliva and reacts with bacteria-derived hydrogen peroxide in reactions involving salivary peroxidases. Hypothiocyanate is finally converted to hypothiocyanite, which inhibits several bacterial glycolytic enzymes, potentially leading to reduced bacterial acid production [67]. Lastly, some facultative bacteria, such as non-mutans streptococci can produce hydrogen peroxide via catalysis of NADH oxidase and pyruvate oxidase [68]. Hydrogen peroxide is cytotoxic and acts as a strong oxidizer in both the host and in bacteria, but some oral bacteria possess catalases and peroxidases, while host tissues possess catalases, which are used to remove hydrogen peroxide. However, the relative role of these oxidative activities in maintaining dynamic stability of the microbiota needs further clarification.

## Maintaining resilience

Bacteria in the oral cavity can survive on nutrients from saliva, only, but in the presence of mixed substrates, carbohydrates are always metabolized first, followed by degradation of proteinaceous substrates [69]. This is a typical condition in the oral cavity, in which overload of carbohydrates from food and drink often results in excess production of organic acids and rapid reduction of environmental pH to <5.5 in the supragingival plaque. This acidification may disturb the mineral balance at the plaque-tooth interphase temporarily, but the intrinsic resilience factors can neutralize acidic pH following eating and drinking. Saliva can wash out carbohydrates and organic acids, neutralize environmental pH via the salivary bicarbonate system, and promote calcium/phosphate reprecipitation in dental plaque and in demineralized dental hard tissues, as long as the duration and frequency of acidification are not high. Similarly, in gingivitis the flow of GCF has a washing and pH-neutralizing function in the gingival crevice because the flow of GCF is positively correlated with inflammation [70,71].

In addition to intrinsic resilience factors, the various pH modulatory bacterial resilience factors described in Figure 2 play an essential role in restoring and balancing pH after disturbance. An ecosystem that does not possess sufficient diversity of functional responses to environmental change cannot be stabilized, and may be subject to regime shift. Molecular microbiological studies have demonstrated that the ‘healthy’ oral microbiota has high species diversity [72,73] suggesting that oral microbial communities generally contain a large pool of functional resilience factors. However, if several microbial species in a community perform similar functions, such as saccharolytic or proteolytic activities, there might be functional redundancy [18]. In such cases, adding more species with the same functional profile, may not improve the functional response to external disturbance. Moreover, if added redundant species do not contribute to additional response diversity it will not be possible to further modulate the community performance. It is therefore important to understand the basic principles of resilience since they might help to explain why ecological approaches to oral disease control, such as increased base production through arginine supplementation [74] or enrichment by probiotic bacteria [75,76] may not turn out as successfully as expected. The former might be due to shortage of arginine-degrading bacteria, while the latter might be due to lack of niches or lack of probiotic species suitable for colonization.

## Restoring resilience

Ecological research states it is relatively easy to break down resilience, whereas it may be difficult to restore resilience [18]. Regime shifts in ecosystems occur mostly as a consequence of human actions. In the oral cavity, breakdown of resilience happens because of changes in the emission of microbial waste products in response to intensified exposure to dietary carbohydrate and/or proteinaceous nutrients in GCF and inflammatory substrates. Initially, such processes are reversible. However, under certain circumstances external drivers may push the ecological processes up to a point – the tipping point – at which the processes become irreversible [77]. This could have dramatic consequences, particularly if the ecosystem suffers from loss of biological and functional sources that allow for renewal and reorganization.

Ecosystem renewal may be particularly difficult in the oral cavity because the mouth contains various econiches, such as teeth, gingival pockets and soft mucosal surfaces, including the tongue, all of which are connected and shed their bacteria into saliva. The shared pool of bacteria in saliva is likely to mirror current disease status [78,79], which could explain why *de novo* biofilm build up on tooth surfaces has been shown to differ between caries-active and caries–inactive individuals [80]. In order to be successful in restoring the resilience of microbial communities on teeth, it may therefore be necessary to restore the functional balance of the microbiota in the entire mouth. Unfortunately, there is limited scientific evidence that this goal is easy to achieve. For example, 2 weeks of daily oral hygiene does not restore the supragingival microbial community profile on teeth to baseline levels following experimental gingivitis development, in spite of prior professional tooth cleaning [81]. Similarly, non-surgical periodontal therapy reduces, but does not eliminate ‘periodontal pathogens’ over 2 years of treatment [82]. Collectively, these observations imply that renewal and reorganization of the microbiota at distinct oral sites is likely to fail unless a holistic approach targeting the primary causes driving breakdown of resilience is taken into account.

The implications of the above reflections are severe because they imply that commonly recommended preventive measures against dental diseases such as tooth cleaning may be inadequate for disease control [83,84] unless combined with control of the ecological drivers of disease, such as fermentable carbohydrates and GCF/inflammatory exudates. For example, it has been suggested that imbalances in the host-immune homeostasis may need to be solved by therapeutic agents mediating resolution of inflammation [10,35]. The key management of dental caries lies in behavioral change advising people to consume a balanced diet low in dietary sugars [85]. This is true, even if the efficacy of dietary interventions may be limited because of poor compliance [86]. Therefore, regular plaque control using a fluoridated toothpaste is mandatory as long as people wish to enjoy the pleasures of a modern diet [87].

## Fermentable carbohydrates – the common risk factor for caries and periodontal diseases?

Ten years ago, Hujoel [88], revisited a long-forgotten hypothesis launched by Cleave [89], and Yudkin [90], in which the authors proposed that a diet rich in fermentable carbohydrates may be the common risk factor for dental and systemic diseases. While cariologists can easily endorse a link between fermentable carbohydrates and caries [91] it may be more difficult to defend a link between fermentable carbohydrate and gingivitis and periodontal diseases. However, Hujoel [88] contends that fermentable carbohydrate may be the ‘forgotten’ cause of periodontal diseases. One of his very strong arguments is that even moderate reduction of carbohydrate in the diet has been shown to reduce gingivitis scores in several clinical trials [88]. Moreover, Baumgartner and co-workers [92] observed that elimination of refined sugars from the diet (‘Stone Age diet’) over a period of 4 weeks without oral hygiene reduced the severity of gingival inflammation (bleeding on probing), in spite of increases in plaque scores. Recently, a reduction of gingival bleeding scores was reported in response to eating an anti-inflammatory diet low in processed carbohydrates and animal proteins [93].

Epidemiological studies provide further evidence that dietary sugars may play a role in periodontal diseases. For example, a large-cross-sectional study of young adults showed that a high consumption of added sugars was associated with periodontal disease, independent of other risk factors, suggesting that sugar consumption and postprandal hyperglycaemia contribute to systemic inflammation in periodontal diseases and other noncomminicable diseases [94]. Another epidemiological study revealed that dental caries and periodontal diseases are associated with each other and with added sugar consumption, obesity, and systemic inflammation [95].

It is well known that hyperglycaemia in diabetes causes inflammatory damage to human tissues, in particular the capillary endothelial cells [for review, 96,97]. The reason why endothelial cells are particularly vulnerable to hyperglycaemia is that these cells are defective in transporting glucose over the cell membrane, which leads to toxic concentrations of glucose inside the cell. This condition evokes a series of host intracellular metabolic events, such as increased susceptibility to oxidative stress and production of advanced glycosylation end-product (AGE) precursors all of which can lead to inflammation and microvascular pathology [96,97]. Similar host metabolic pathways have been described in gingival tissues of gingivitis and periodontitis [for review, 98,99]. It has therefore been speculated that gingivitis and periodontitis are typical sugar-driven inflammatory diseases, similar to type 2 diabetes, obesity, and cardiovascular diseases [93,100] and not just corollaries to systemic diseases [99,101,102].

The above reflections gain further support from studies of experimental gingivitis in which it was shown that type 1 diabetics developed an earlier and higher inflammatory response than non-diabetics to a comparable bacterial challenge [103–105,]. Moreover, a 5-year-prospective study of periodontitis progression in diabetic patients concluded that it is the glycotoxicity of hyperglycaemia in diabetes rather than the type of diabetes *per se*, that is the aberrant factor in periodontal breakdown [106]. Only poorly controlled diabetes caused periodontitis progression during the follow-up period. Hence, hyperglycaemia may have a direct inflammatory effect on the periodontal tissues. An alternative indirect scenario could be that hyperglycaemia-affected periodontal tissues are more vulnerable to bacterial metabolites and elicit a higher GCF response than unaffected tissues. However, we do not know if increased GCF secretion is one of the characteristics of tissue inflammation or if it mainly represents a response to bacterial load.

It may be argued that in this discussion we overestimate the role of fermentable carbohydrates for the development of periodontal diseases. While it is true that patients with a high carbohydrate intake may experience periodontitis, this may not be the case for all patients since there are other patients with low carbohydrate intake, yet still manifest with periodontitis. On the contrary, there are patients who have a diet rich in carbohydrate, yet very little caries and periodontal disease experience. In this context, it should be appreciated that dental caries and periodontal diseases are multifactorial disorders [107,108]. Microbial deposits on teeth is the only necessary causal factor for the development of these diseases, whereas environmental factors (such as diet, smoking and fluoride exposure), host factors (such as host immune and inflammatory responses), and systemic factors (such as diabetes and chronic inflammatory conditions) are modulatory factors in the disease processes [10,109]. It is the interplay between these and numerous other risk factors (known and unknown) including intrinsic risk factors such as saliva and GCF that determines the outcome of caries and periodontal diseases in the individual patient. Furthermore, because of their multifactorial nature, these diseases are not mutually exclusive. Hence, it is not surprising that a susceptible host with a weak host response and infrequent exposure to fluoride by tooth cleaning may suffer both caries and periodontal disease in the presence of a carbohydrate-rich diet. Conversely, in another patient the deleterious effects of high carbohydrate intake might be alleviated by other risk factors.

## Conclusion

This review supports an integrated hypothesis for dental caries and periodontal diseases that builds on sound theoretical ecological principles. The backbone of the hypothesis is based on the dynamic stability stage of the oral microbiota, at which the intrinsic (mainly saliva and GCF) and bacterial (mainly metabolic) resilience factors maintain ecological dynamic stability, compatible with clinical health. However, loss of intrinsic resilience factors and/or prolonged changes in the availability of microbial metabolic substrates may shift the ecological balance of the microbiota into either saccharolytic (acidogenic) or amino acid-degrading/proteolytic (alkalinogenic) stages, depending on the nature of the predominant substrates (fermentable carbohydrate or GCF/inflammatory exudate), leading to clinical diseases. Therefore, to maintain and restore the dynamic stability of the oral microbiota, it is necessary to control the drivers of disease, such as the influx of bacterial nutrients into the oral cavity. It is better to focus on maintenance of resilience of the oral microbiota than on its restoration, because restoration of resilience might take years unless key ecological factors (such as sugar consumption and GCF/inflammation) are controlled.

The integrated hypothesis of dental caries and periodontal diseases proposed in this review is entirely compatible with the Cleave-Yudkin hypothesis assuming that excess intake of fermentable carbohydrate is a shared risk factor of bacterially induced dental diseases. While the pathophysiology and tissue responses differ – demineralization of dental hard tissues in caries, and inflammation in gingivitis and periodontitis – both diseases originate in the dynamic stability stage and emerge in response to nutritional imbalances in the microbiota. Future studies should investigate the pathogenic mechanisms linking sugar consumption and periodontal diseases and explore to which extent these diseases may be controlled by stabilization of blood sugar levels. This research question is not only of theoretical importance. If it can be confirmed that fermentable carbohydrate has been overlooked as a risk factor for the development of inflammation in periodontal tissues, this should lead to radical changes of the guidelines for periodontal disease control in the future.
